# Ropivacaine promotes apoptosis of hepatocellular carcinoma cells through damaging mitochondria and activating caspase-3 activity

**DOI:** 10.1186/s40659-019-0242-7

**Published:** 2019-07-12

**Authors:** Wenting Wang, Mingyue Zhu, Zhixin Xu, Wei Li, Xu Dong, Yi Chen, Bo Lin, Mengsen Li

**Affiliations:** 1 Hainan Provincial Key Laboratory of Carcinogenesis and Intervention, Hainan Medical College, 3 Xueyuan Road, Longhua District, Haikou, 571199 Hainan People’s Republic of China; 2 Department of Anaesthesiology, Second Affiliated Hospital, Hainan Medical College, Haikou, 570311 Hainan People’s Republic of China; 3 Key Laboratory of Molecular Biology, Hainan Medical College, Haikou, 571199 People’s Republic of China; 4 Institution of Tumour, Hainan Medical College, Haikou, 570102 Hainan People’s Republic of China

**Keywords:** Local anaesthetic agent, Apoptosis, Hepatocellular carcinoma cells, Signal transduction, Caspase-3 activity

## Abstract

**Background:**

Recent evidences indicated that some local anaesthetic agents played a role in inhibiting the proliferation of cancer cells; Whether ropivacaine is able to promote apoptosis of hepatocellular carcinoma (HCC) cells is still unclear. The aim of this study was to investigate the effect of ropivacaine on the apoptosis of HCC cells.

**Methods:**

In the present study, we treated the HCC cell lines, Bel7402 and HLE with ropivacaine. MTT, DAPI stain, trypan blue exclusion dye assay, flow cytometry, electron microscopy, computational simulation, laser confocal microscope, Western blotting, and enzyme activity analysis of caspase-3 were applied to detect the growth and apoptosis of HCC cells and to explore the role mechanism of ropivacaine.

**Results:**

Ropivacaine was able to inhibit proliferation and promote apoptosis of HCC cells in a dose- and time-dependent manner. Ropivacaine also has a trait to inhibit the migration of HCC cells; ropivacaine damaged the mitochondria of HCC cells. The results also indicated that ropivacaine was able to interact with caspase-3, promote cytoplasmic caspase-3 migration into the nucleus, stimulate cleavage of caspase-3 and PARP-1, caspase-9 proteins, inhibit the expression of Bcl-2, promote expression of Apaf-1 and mitochondria release cytochrome C, and activate the activity of caspase-3.

**Conclusions:**

Ropivacaine has a novel role in promoting apoptosis of HCC cells; The role mechanism of ropivacaine maybe involve in damaging the function of mitochondria and activating the caspase-3 signalling pathway in HCC cells. Our findings provide novel insights into the local anaesthetic agents in the therapy of HCC patients.

**Electronic supplementary material:**

The online version of this article (10.1186/s40659-019-0242-7) contains supplementary material, which is available to authorized users.

## Background

Local anaesthetic agents have broad therapeutic functions that go beyond the known analgesia and antiarrhythmic [1]. They are applied in a wide range of clinical situations to prevent or reduce acute pain, chronic pain and cancer pain [2, 3]. A retrospective analysis of patients undergoing cancer surgery suggests that using local anaesthesia may reduce cancer recurrence and improve survival rate [3, 4]. Recently, some documents have shown that local anaesthetic agents inhibit proliferation, invasion and migration, and promote apoptosis at a range of specific concentrations [5–7]. Lidocaine and ropivacaine effectively inhibit the invasiveness of lung cancer and colon cancer cells at specific concentrations; The anti-invasive effect seems unrelated to its anaesthetic activity (sodium channel blockade) but related to the cellular signal transduction, such as the Akt signal pathway [8–10]. Other local anaesthetics have been shown to stimulate apoptosis in a variety of cancer cells [11–13]. The mechanisms involved in these effects are not yet fully understood. Recent evidences indicated that lidocaine played a role in anti-proliferation and induced apoptosis of hepatocellular carcinoma (HCC) cells [14, 15], which implicated that local anaesthetics can suppress the growth of HCC cells.

Ropivacaine is widely used for interventional spinal procedures and is generally accepted as safe. Recently, several studies have focused on the potential cytotoxic effects of ropivacaine on HCC cells [14, 15]. The evidence from these studies showed that ropivacaine inhibits the viability of HCC cells in a dose- and time-dependent manner. However, the underlying mechanism of how ropivacaine induces HCC cells death has not been fully elucidated. In the present study, we investigated the effects of ropivacaine on the proliferation and apoptosis of the human HCC cells lines, Bel 7402 and HLE. We found that ropivacaine was capable of inhibiting viability, stimulating apoptosis of HCC cells, damaging mitochondria, and activating the activity of caspase-3 in HCC cells.

## Material and methods

### Cell culture

In this study, we selected the human HCC cell lines, Bel 7402 and HLE for testing. These cells were gifts from the Department of Cell Biology, Peking University Health Science Centre (Beijing, China); The cells were cultured in RPMI-160 medium supplemented with 10% heat-inactivated foetal calf serum (FCS) and were incubated at 37 °C in a humidified atmosphere containing 5% CO_2_ as previously described [16].

### Cell growth detection by MTT methods

A total of 1.5 × 10^4^ Bel 7402 or HLE cells were plated in each well of 96-well plates and cultured in RPMI-1640 medium supplemented with 10% FCS at 37 °C in a humidified atmosphere of 5% CO_2_ for 48 h then treated with different concentrations of ropivacaine (0.25–4.0 mmol/L) for 24 h, 48 h, and 72 h. The effects of ropivacaine (Sigma-Aldrich Company Ltd, St. Louis, MO, USA) on the growth of HCC cells were measured by a methylthiazolyldiphenyl-tetrazolium bromide (MTT) assay as previously described [17].

### Cell morphology was observed by microscopy and nuclear staining with DAPI

To observe alterations in cellular morphology that induced by ropivacaine, Bel 7402 cells or HLE cells were plated at a density of 2.0 × 10^4^/mL in 24-well plates. The cells were cultured in RPMI-1640 medium supplemented with 10% FCS at 37 °C in a humidified atmosphere of 5% CO_2_ for 48 h then treated with 0.5 mmol/L, 1.0 mmol/L or 2.0 mmol/L of ropivacaine. After treatment for 48 h, cellular morphology was observed under light microscopy, and the cells were stained with 4,6-diamidino-2-phenylindole dihydrochloride(DAPI) solution. The cells were imaged using a fluorescent microscope at 100× magnification. In this study, nuclear pyknosis and fragmentation were taken to support evidence of apoptosis, and these criteria were evaluated by fluorescent microscopy as previously described [18, 19].

### Trypan blue exclusion dye method to analyse cells viability and metabolic activity

To determine cellular viability, Bel 7402 cells or HLE cells were seeded at a density of 2.5 × 10^4^ cells per well in 6-well plates, the cells were cultured in RPMI-1640 medium supplemented with 10% FCS at 37 °C in a humidified atmosphere of 5% CO_2_ for 48 h. Following treatment with different concentrations (0.5–2.0 mmol/L) of ropivacaine for 48 h, cellular viability was determined by trypan blue exclusion dye assay using a Trypan Blue Staining Cell Viability Assay Kit (Beyotime Biotech Corp, Haimen, Jiangshu, China). Cells restricting trypan blue entry were considered viable; Cellular viability ratio = (control group viable cells-treated groups viable cells)/control group viable cells × 100%.

### Flow cytometry was used to analyse apoptosis

Bel 7402 cells and HLE cells were cultured in RPMI-1640 medium supplemented with 10% FCS at 37 °C in a humidified atmosphere of 5% CO_2_. The cells were treated with ropivacaine (2.0 mmol/L) for 48 h, and the apoptosis in Bel 7402 cells or HLE cells was analyzed by flow cytometry as previously described [17].

### Electron microscope observation

Bel 7402 cells and HLE cells were cultured in RPMI-1640 medium supplemented with 10% FCS at 37 °C in a humidified atmosphere of 5% CO_2_. The cells were treated with ropivacaine (2.0 mmol/L) for 48 h, and then the cells were harvested, washed with PBS(phosphate buffer saline) solution two times (4 °C, 10 mL for 8 min), and transferred to 1.5 mL EP tubes. The cells were washed with PBS three times (15 min/time) and then fixed in 1% osmic acid (60 min) and 2% uranyl acetate (30 min). After dehydration in a gradient ethanol series, the cells were permeated with pure acetone and embedding medium (1:1) for 60 min, and then permeated with embedding medium alone for another 60 min. The samples were dried in an oven at 37 °C for 24 h, 45 °C for 24 h and 60 °C for 48 h. Ultrathin sections (0.1 μM) were prepared and examined under a TECNA 10 transmission electron microscope (Philips, Holland). Mitochondria were observed by randomly selecting ten cells from each group.

### Sequence alignment, molecular modelling, docking and simulation

Caspase-3 was modelled as previously described [20], and the following structural files were obtained from the protein data bank (PDB): caspase-3 (PDB: 1CP3). Caspase-3 modelling was performed using Modeller version 9.0, and docking was achieved using ZDOCK. Ropivacaine was used for all simulations.

### Laser confocal microscope observation

Bel 7402 cells and HLE cells were stained as described previously [21]. Briefly, cells were fixed in 4% paraformaldehyde and incubated with mouse anti-human caspase-3 antibody for 12 h. Fluorescein isothiocyanate (FITC)-conjugated secondary anti-mouse immunoglobulin G was added and incubated for 2 h, followed by the addition of 100 μL DAPI (1 μg/mL) for 30 min. Cells were visualized with a Leica TCS-NT SP2 laser confocal microscope (Leica Camera, Wetzlar, Germany).

### Western blotting analysis

To estimate the influence of ropivacaine on the expression of cytoplasmic apoptosis-related proteins and mitochondria apoptosis-related proteins, Bel 7402 cells and HLE cells were treated with ropivacaine (2.0 mmol/L) for 24 h, and the expression of apoptosis-related proteins, such as activated caspase-3, activated PARP-1, Bcl-2, Apaf-1, cleaved-caspase-9 and Cytochrome C in Bel 7402 cells or in HLE cells were analyzed by Western blotting as previously described [17].

### Analysis the activity of caspase-3

Bel 7402 cells and HLE cells were treated with ropivacaine (2.0 mmol/L) or caspase-3 inhibitor (Z-DEVD-FMK)(Selleck Chemicals Company, USA) 1.0 μmol/L for 24 h. Caspase-3 activity was measured with a commercial kit according to the manufacturer’s protocols (APOPCYTO Caspase-3 Colorimetric Assay Kit; Medical and Biological Laboratories, Japan) as described in a previous study [21].

### Statistical analysis

The data are presented as the mean ± S.D. The statistical analysis was performed using Student’s t test (for two experimental groups). The significance was set at *P* < 0.05. Statistical significance was determined using Student’s t-test and *F* test (SPSS 11.5 software for Windows, SPSS Inc., Chicago, IL, US).

## Results

### Ropivacaine inhibited the growth of HCC cells

**Fig. 1:**
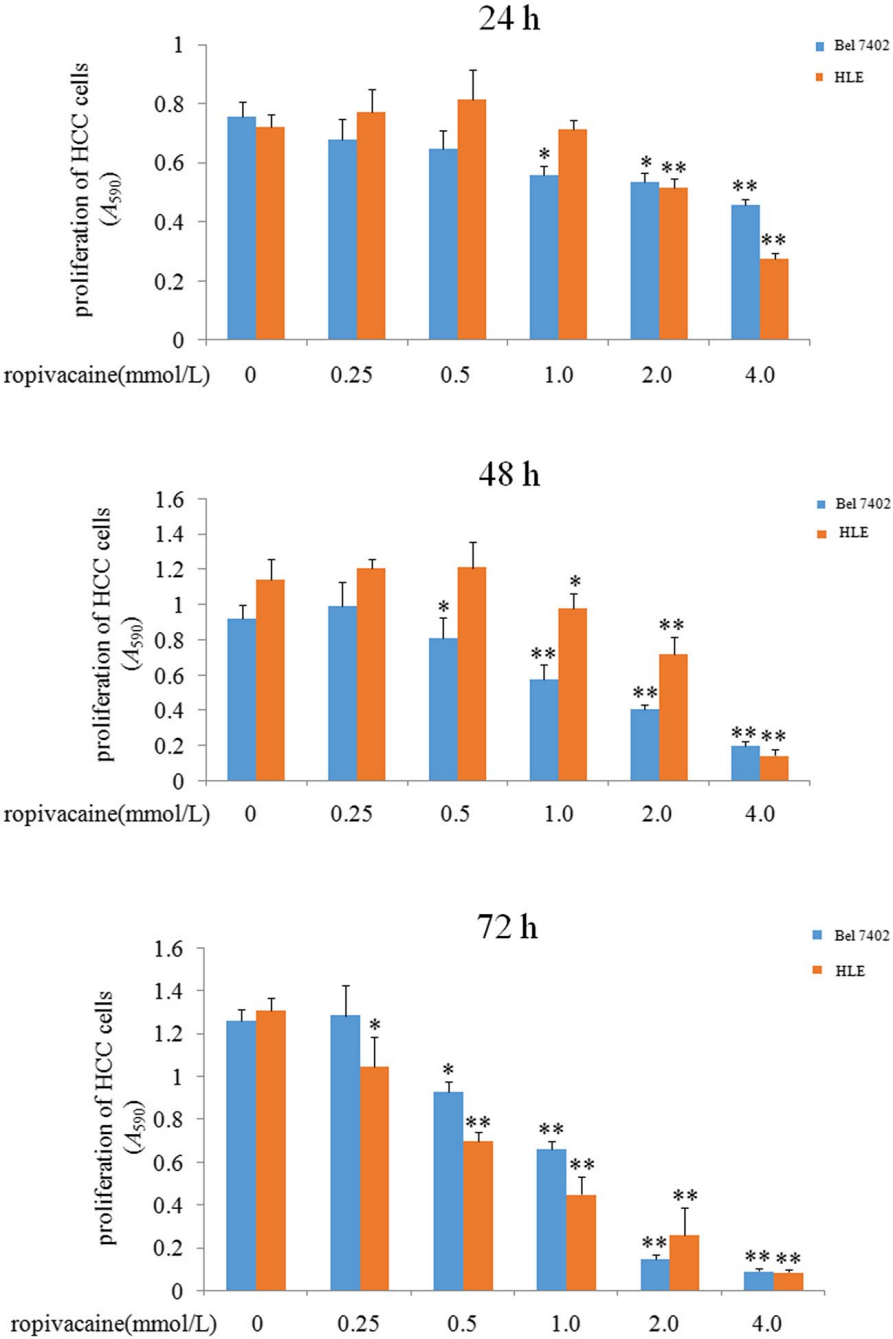
Influence of ropivacaine on the growth of Bel 7402 and HLE cells. Bel 7402 cells and HLE cells were treated with different concentrations (0.25 mmol/L, 0.5 mmol/L, 1.0 mmol/L, 2.0 mmol/L and 4.0 mmol/L) of ropivacaine for 24 h, 48 h and 72 h. The MTT assay was applied to detect the growth of the cells. *P < 0.05 and **P < 0.01 vs control groups (0 mmol/L). N = 6

In the study, we treated with human hepatoma cell lines, Bel 7402 and HLE of difference concentrations of ropivacaine (0.25, 0.5, 1.0, 2.0 and 4.0 mmol/L) for 24, 48, 72 h respectively, and then the MTT assay was applied to detect the growth of these HCC cells. The results indicated that ropivacaine was able to inhibit the growth of HCC cells in a dose- and time-dependent manner (Fig. 1). The results displayed that the concentration > 1.0 mmol/L of ropivacaine was significantly inhibited the proliferation of HCC cells.

### Ropivacaine promotes apoptosis of HCC cells

**Fig. 2:**
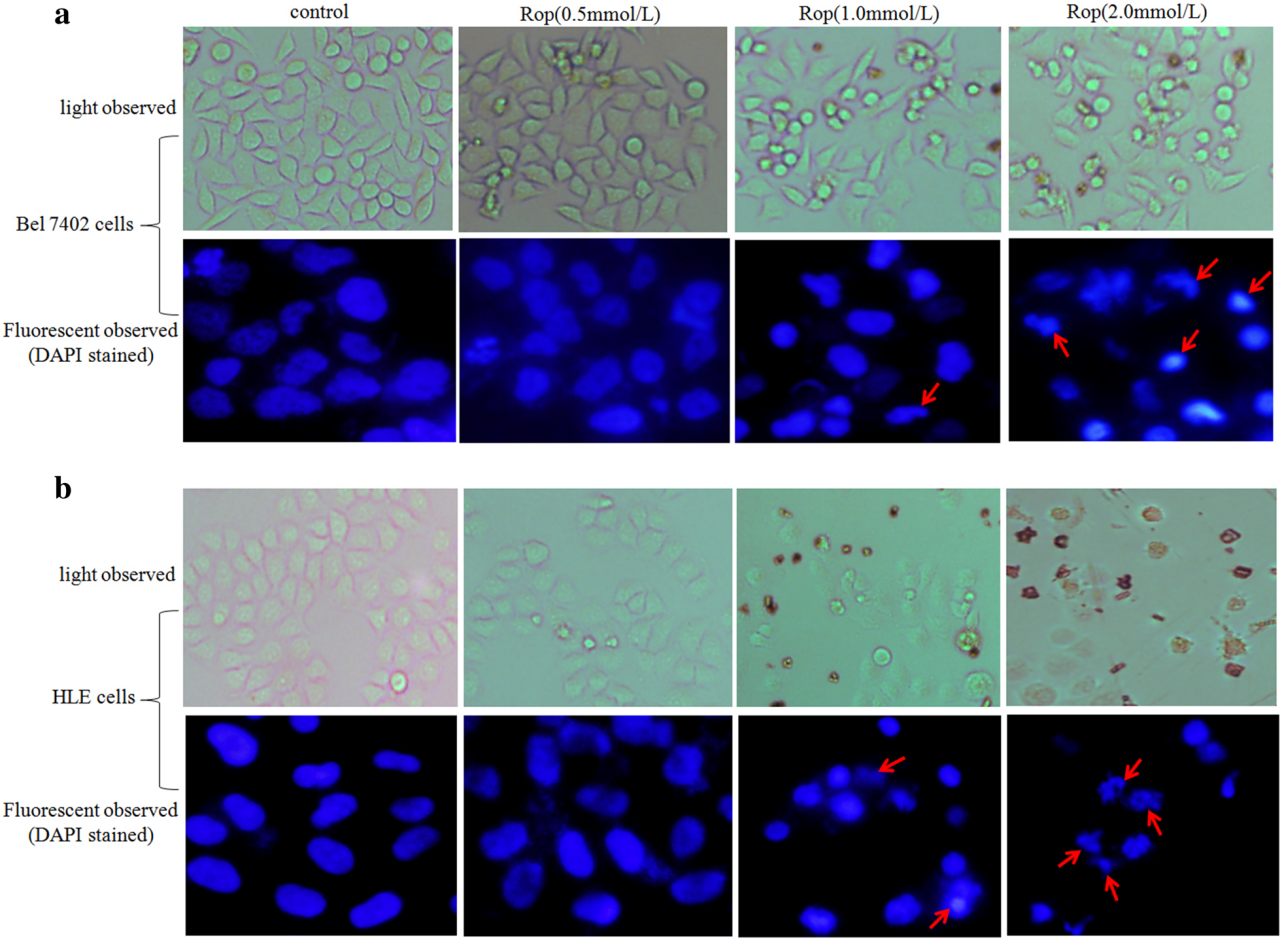
Influence of ropivacaine (Rop) on the genesis of apoptosome in Bel 7402 cells and HLE cells. Bel 7402 cells (**a**) and HLE cells (**b**) were treated with (2 mmol/L) of Rop for 48 h, the cellular morphology of Bel 7402 cells or HLE cells was observed by microscopy. The cytoblasts of Bel 7402 cells and HLE cells were stained with DAPI and observed by fluorescence microscopy. The red arrows indicate apoptosomes. The images are representation of at least three independent experiments

**Fig. 3:**
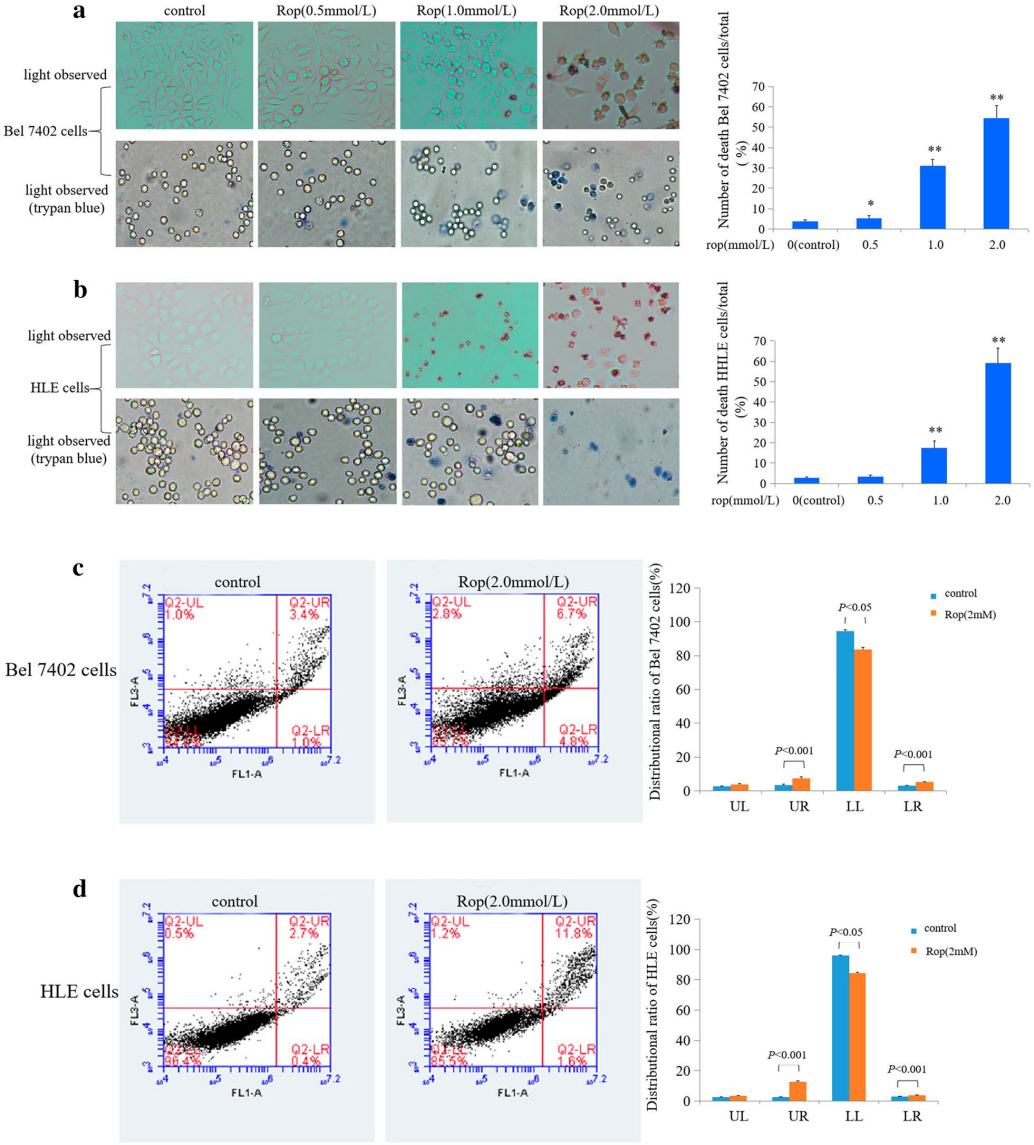
Influence of Rop on Bel 7402 cells and HLE cells apoptotic ratio. Bel 7402 cells (**a**) and HLE cells (**b**) were treated with the different concentrations (0.5 mmol/L, 1.0 mmol/L, 2.0 mmol/L) of Rop for 24 h. Trypan blue exclusion dye assay was used to analyse the apoptotic ratio of the cells. The images were observed by microscope, and the right columnar graph shows the statistical value of apoptotic ratio. *P < 0.05 and **P < 0.01 vs control group; N = 6. Bel 7402 cells (**c**) and HLE cells (**d**) were treated with 2 mmol/L of Rop for 48 h, and the apoptosis of Bel 7402 cells and HLE cells was analysed by flow cytometry. The right columnar graph shows the statistical analysis of the apoptosis ratios; *P < 0.05, **P < 0.01 vs. control groups. The images are a representation of at least three independent experiments

To explore the effect of ropivacaine on the apoptosis of HCC cells, in the present investigation, Bel 7402 cells and HLE cells were treated with different concentrations of ropivacaine (0.5, 1.0, 2.0 mmol/L) for 48 h. We performed cell morphological observations. Figure 2a, b showed that morphological changes occurred in Bel 7402 cells and HLE cells while treated with ropivacaine (Rop, 1.0, 2.0 mmol/L). Nuclear morphology changes were observed in Bel 7402 cells and HLE cells under the fluorescence microscope using DAPI staining. The results revealed that Rop also induced apoptosome occurrence in the Bel 7402 cells and HLE cells. Cellular nuclear condensation and pyknosis were significantly increased, and morphological characteristics of apoptosis, including apoptosome formation and nuclear shrinkage, were apparent in the Rop-treated (1.0, 2.0 mmol/L) Bel 7402 cells and HLE cells (Fig. 2a, b). However, few changes were observed in the cells treated with Rop (0.5 mmol/L) or the untreated group. In order to observe the apoptosis of HCC cells, in the study, we applied trypan blue exclusion dye to visualize cellular viability and metabolic activity. The results indicated that dead cell numbers significantly increased in the cells while treated with Rop (0.5, 1.0, 2.0 mmol/L) for 48 h compared to the untreated groups (Fig. 3a, b). We also utilized flow cytometry to analyse apoptosis of HCC cells, the results revealed that apoptosis of Bel 7402 cells and HLE cells were significantly increased in the cells while treated with Rop (2.0 mmol/L) for 48 h compared to the untreated groups (Fig. 3c, d). These results indicated that Rop has a trait to promote apoptosis of HCC cells.

### Ropivacaine damaged the function of mitochondria of HCC cells

**Fig. 4:**
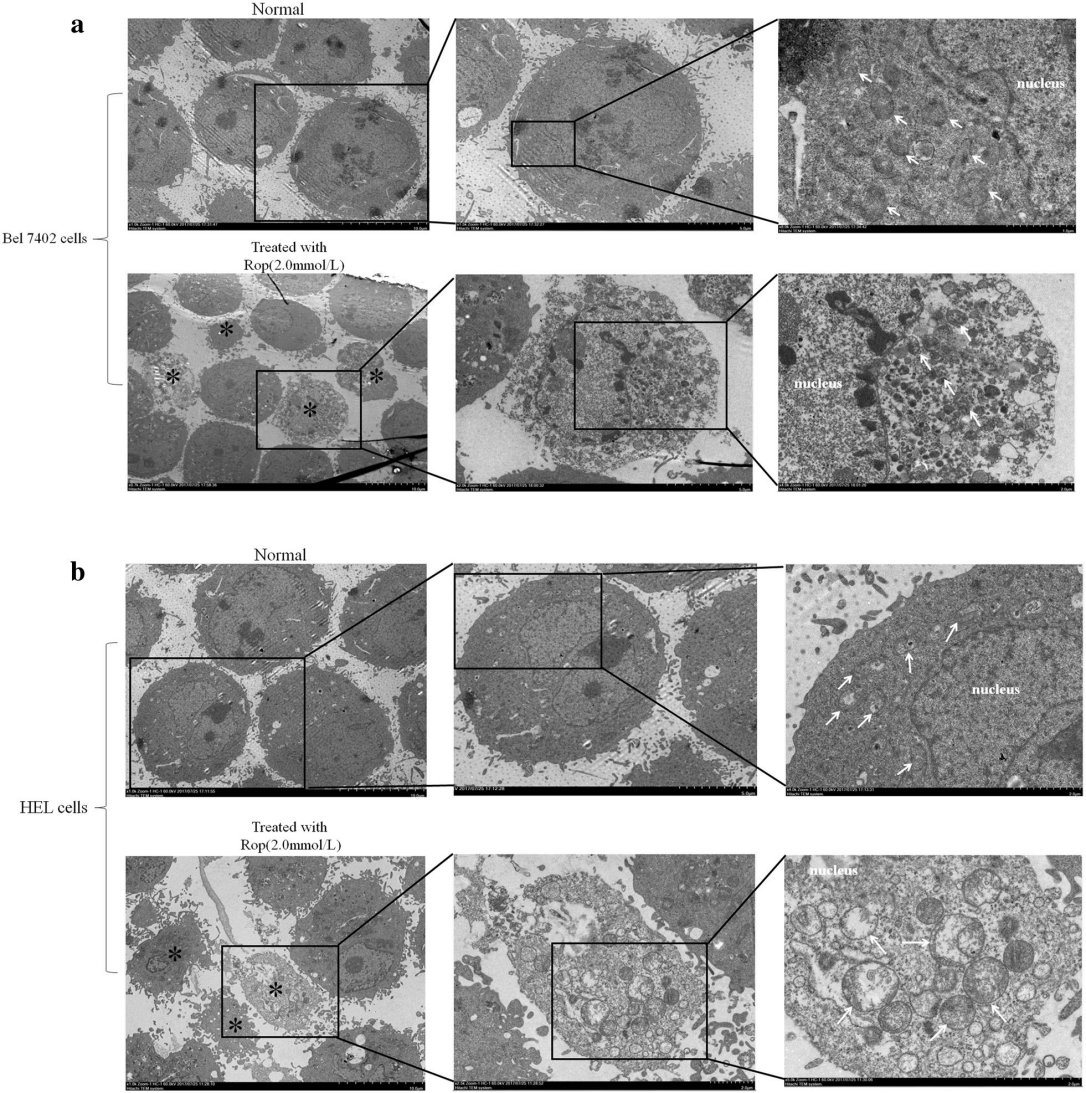
Effects of Rop on mitochondria function of Bel7402 cells and HLE cells. Bel 7402 cells (**a**) and HLE cells (**b**) were treated with Rop (2.0 mmol/L) for 24 h, the morphological change of mitochondria of the cells was observed by electron microscope. * Indicates the apoptotic cells, and white arrows show the mitochondria. The images represent three independent experiments

To explore the role mechanism of Rop on stimulating apoptosis of HCC cells, in the present study, we used electron microscopy to observe the structural changes of mitochondria in HCC cells. The results indicated that when the cells were treated with Rop (2.0 mmol/L) for 48 h, mitochondria swelling and ridge breakage occurred in the apoptotic HCC cells (Fig. 4a, b). These results demonstrated that Rop was able to damage the mitochondria function in HCC cells.

### Ropivacaine stimulated activity of caspase-3 and promoted expression of aopoptosis-related proteins in HCC cells

**Fig. 5:**
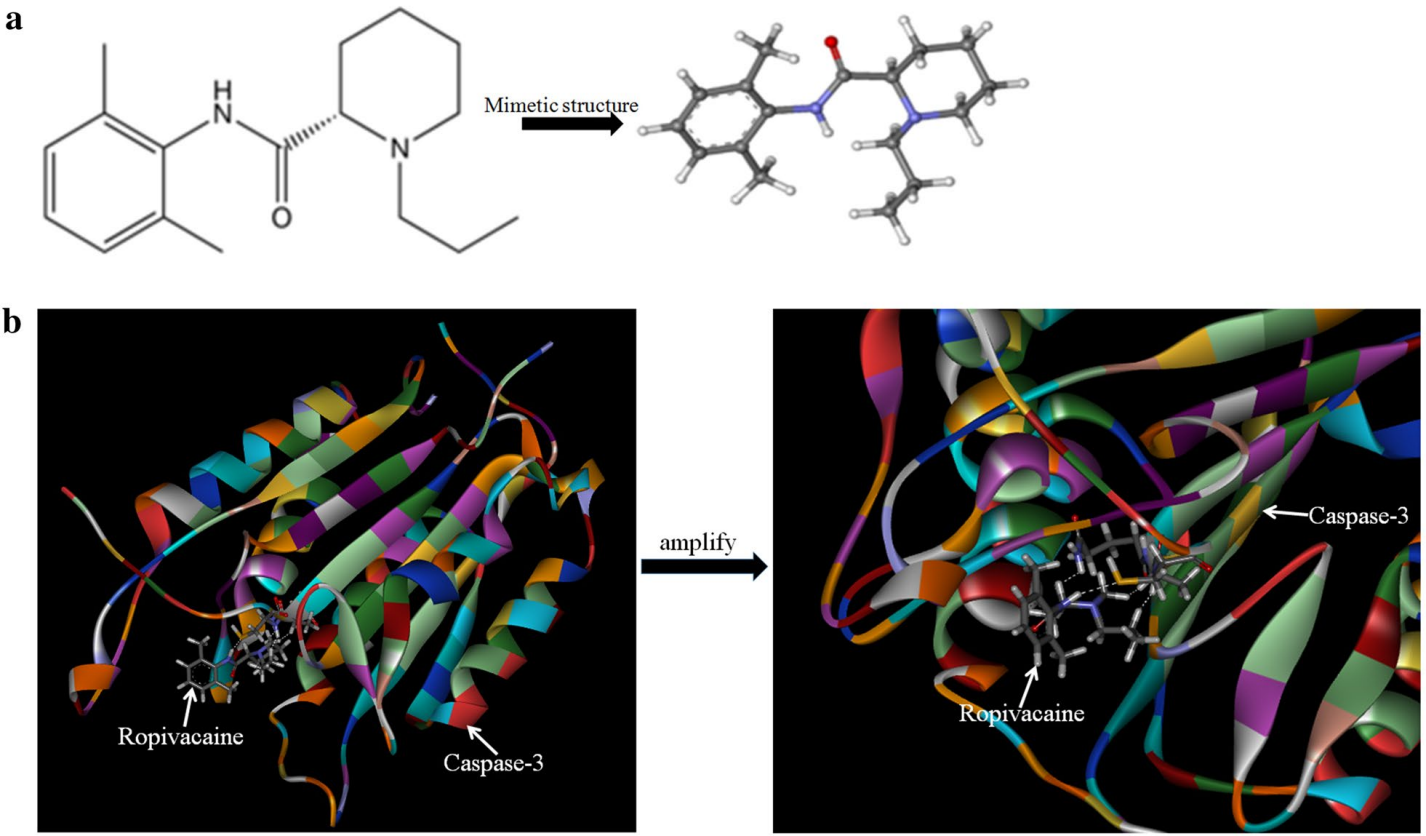
Modelling simulation of Rop binding to caspase-3. **a** Rop chemical structure. **b** Rop binding to caspase-3 using the model

**Fig. 6:**
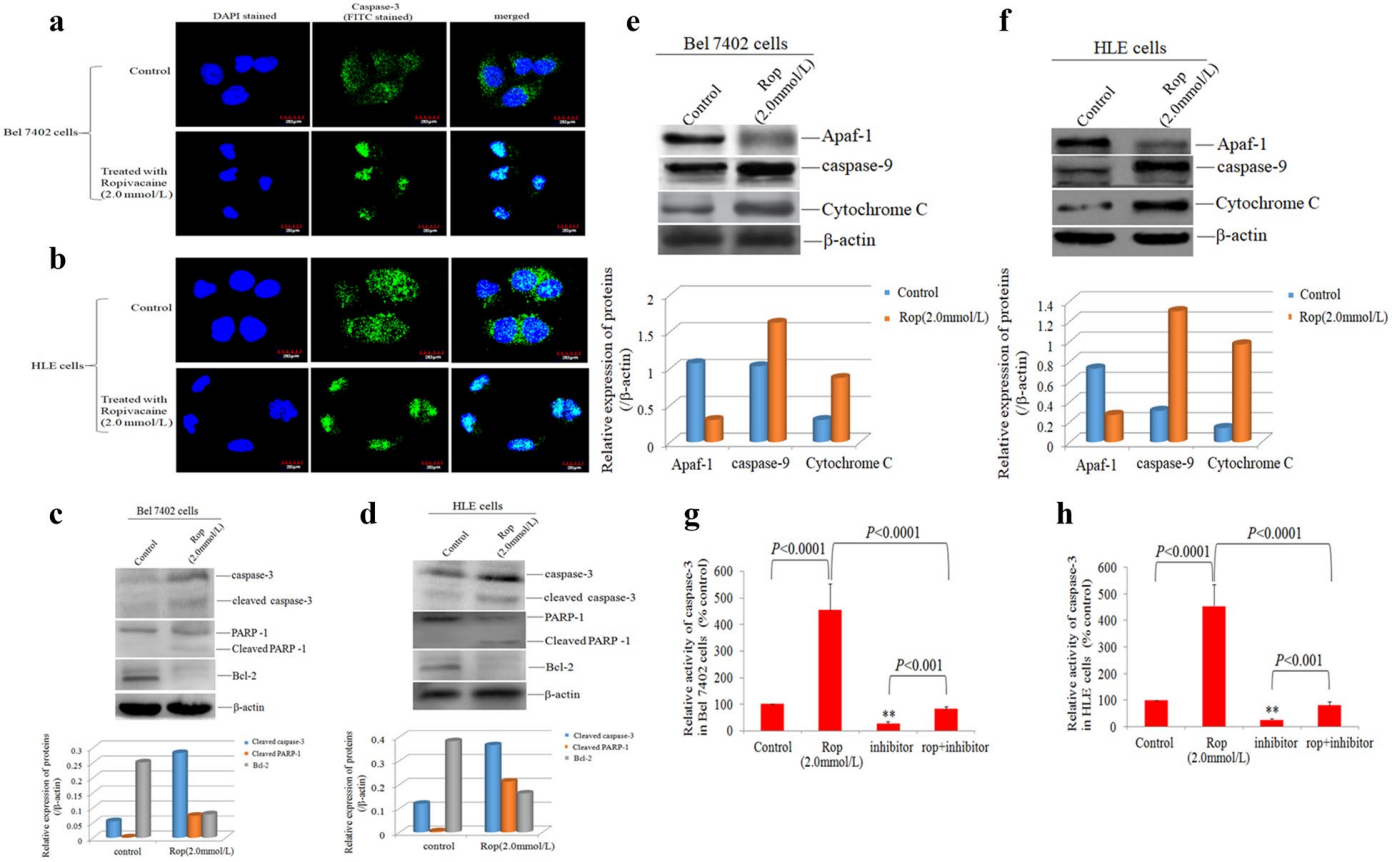
Effects of Rop on the nucleus migration and the activity of caspase-3, the expression of apoptosis-related proteins in HCC cells. Bel 7402 cells (**a**) and HLE cells (**b**) were treated with Rop (2.0 mmol/L) for 24 h; the nucleus migration of caspase-3 in Bel 7402 cells and HLE cells were observed by laser confocal microscopy. The expression of caspase-3, activated caspase-3 (cleaved), PARP-1, activated PARP-1 (cleaved-PARP-1) and Bcl-2 (**c**, **d**). The expression of Apaf-1, caspase-9 (cleaved) and cytochrome C (**e**, **f**) in Bel 7402 cells and HLE cells were analysed by Western blotting. The bottom columnar graph depicts the statistical analysis of the relative expression of these proteins. The activity of caspase-3 in Bel 7402 cells (**g**) and HLE cells (**h**) was measured using an enzymatic reaction reagent kit. **P < 0.01 vs control group, N = 6. The images represent at least three independent experiments

In the present study, we applied sequence alignment, molecular modelling, docking and simulation to analyse the interaction of Rop and caspase-3. The results indicated Rop binding with the caspase-3 catalytic structure domain (Fig. 5a, b). Laser confocal microscopy observation showed caspase-3 molecule migration into the nucleus (Fig. 6a, b), and Western blotting analysis indicated increased cleavage of caspase-3 and PARP-1. The expression of Bcl-2 was significantly decreased (Fig. 6c, d), and the expression of Apaf-1, cleaved-caspase-9, cytochrome C were significantly increased (Fig. 6e, f), the activity of caspase-3 was significantly stimulated (Fig. 6g, h) when Bel 7402 cells or HLE cells were treated with Rop (2.0 mmol/L) for 48 h. These results demonstrated that Rop interacted with caspase-3, promoted nuclear migration and activated the activity of caspase-3, Rop was able to promote expression of mitochondria related-apoptosis proteins in HCC cells.

## Discussion

Local anaesthetics are known to promote apoptosis of cancer cells in clinically relevant concentrations. Some studies have suggested that local anaesthetics stimulated apoptosis of cancer cells may be unrelated to the sodium-channel blockade [9], while in other studies have reported that the role of local anaesthetics is to inhibit the transduction of the PI3K/Akt/mTOR signal pathway [11]. Retrospective investigations of patients undergoing cancer surgeries showed that using regional anaesthesia was able to reduce the risk of cancer recurrence and metastasis, but the mechanism remains unclear [22–24]. Although some studies indicated that local anaesthetic agents played a role in inducing apoptosis of cancer cells, it is unknown what determines the apoptotic potency of local anaesthetics. Rop also has the ability to stimulate apoptosis of some cancer cells [6, 11, 25]; However, little is known about Rop induced apoptosis of HCC cells and its role mechanism. Therefore, in this study, we compared apoptosis induction by Rop related to their physicochemical properties in human HCC cells. The results suggested that Rop induces apoptosis of Bel 7402 cells and HLE cells in a dose- and time-dependent manner.

The functional damage of mitochondria may lead to death or apoptosis of cells. Mitochondria provide energy for the growth of cells through promoting the metabolism of carbohydrates. Swelling and ridge breakage indicated the functional loss of mitochondria. In the present study, electron microscopy observation indicated that mitochondrial swelling and ridge breakage emerged in the human HCC cells lines, Bel 7402 and HLE when treated with Rop. These results revealed that Rop promoted apoptosis of HCC cells involved in damaging the structure and inhibiting the function of mitochondria.

Caspase-3 is a critical molecule for stimulating apoptosis of cancer. Cleaved caspase-3, the active form of caspase-3, was the main cleavage enzyme to promote apoptosis [26, 27]. PARP-1 could be cleaved by caspase-3 during apoptosis, which was involved in DNA damage and repair. This cleaved PARP-1 contributed to cell apoptosis [28]; Bcl-2 plays a role in inhibition of caspase-3 activity [29], but Apaf-1 was able to activate caspase-9 [30], cleaved caspase-9 (activated form) and cytochrome C were able to activate caspase-3. In the present investigation, we found that the expression of Apaf-1, cleaved caspase-9, cytochrome C, cleaved caspase-3 and PARP-1 were increased, but the expression of Bcl-2 was decreased, and the activity of caspase-3 was stimulated when HCC cells were treated with Rop. The results also indicated Rop interaction with caspase-3. Some studies showed that Rop inhibited cancer cells growth and survival involved in suppressing expression of cell cycle-related genes and the tranduction of PI3K/Akt/mTOR signal pathway [6, 11]. These results showed that Rop-induced apoptosis of HCC cells was closely related to activation of caspase-3 and damage the function of mitochdria.

## Conclusions

Rop affects the outcomes of HCC cells in a variety of aspects: promoted apoptosis and inhibited growth, invasion and migration (Additional file 1: Figure S1). The study demonstrated that Rop stimulated apoptosis of HCC cells through interacting with caspase-3, inhibiting the activity of caspas-3 and damaging the function of mitochondria. This is the first evidence that Rop is able to bind with the catalytic domain of caspase-3. The present study indicated the additional benefits of Rop in liver cancer surgery, which may have substantial clinical implications. This study provides a novel strategy for using Rop synergy with other anticancer agents for therapy in HCC patients.

## Additional file

**Additional file 1: Figure S1.** Effects of Rop on wound healing of Bel7402 cells and HLE cells. Bel 7402 cells (A) and HLE cells (B) were used in a scratch assay, and the cells were treated with Rop (2.0 mmol/L) for 24 h, 48 h and 72 h. The wound healing of the cells was observed by microscopy; the right columnar graph shows the repair ratio of the cells; *P < 0.05, **P < 0.01 versus control groups (0 mmol/L). The images represent three independent experiments.

## Data Availability

Not applicable.

## Abbreviations

MTT: 3-(4,5-dimethyl-2-thiazolyl)-2,5-diphenyl-2-H-tetrazolium bromide
DAPI: 4′,6-diamidino-2-phenylindole
PARP-1: poly ADP-ribose polymerase 1
Apaf-1: apoptotic protease-activating factor 1
Rop: ropivacaine
