# Supplementary figures and images for: Ropivacaine promotes apoptosis of hepatocellular carcinoma cells through damaging mitochondria and activating caspase-3 activity
Source: Biol Res. 2019 Jul 12;52:36. doi: 10.1186/s40659-019-0242-7 (PMC6625015; doi:10.1186/s40659-019-0242-7)

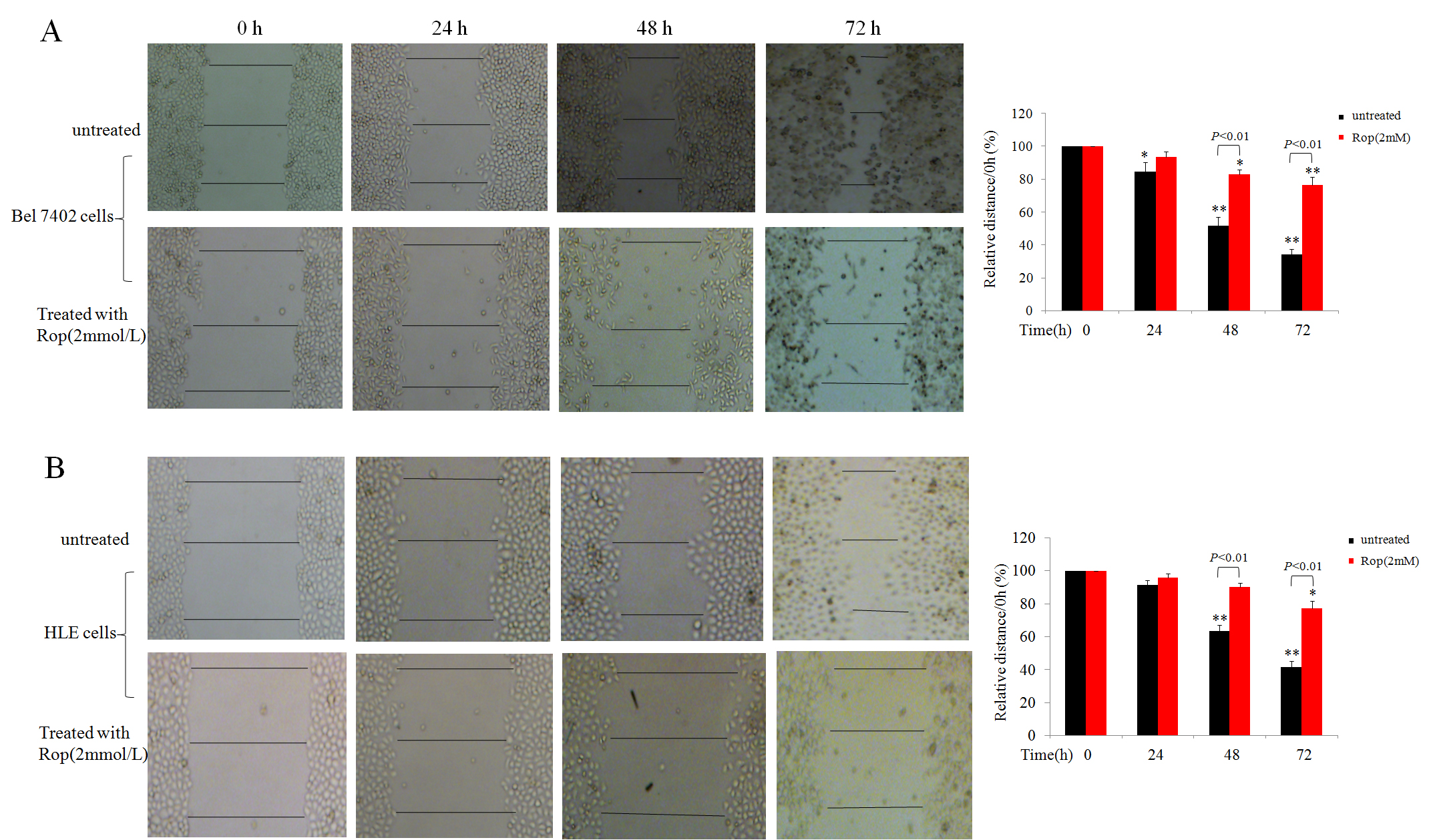

Supplement: Supplementary file 1 — Additional file 1: Figure S1. Effects of Rop on wound healing of Bel7402 cells and HLE cells. Bel 7402 cells (A) and HLE cells (B) were used in a scratch assay, and the cells were treated with Rop (2.0 mmol/L) for 24 h, 48 h and 72 h. The wound healing of the cells was observed by microscopy; the right columnar graph shows the repair ratio of the cells; *P < 0.05, **P < 0.01 versus control groups (0 mmol/L). The images represent three independent experiments. [file 40659_2019_242_MOESM1_ESM.jpg]
